# A176 UTILITY OF MACHINE LEARNING FOR SERUM METABOLOMIC DATA ANALYSIS IN PEDIATRIC CROHN DISEASE

**DOI:** 10.1093/jcag/gwab049.175

**Published:** 2022-02-21

**Authors:** R G Suarez Suarez, S I Dijk, Z Zhang, G Focht, V Navas-López, S Koletzko, A Griffiths, D S Wishart, R Greiner, D Turner, E Wine

**Affiliations:** 1 Paediatrics, University of Alberta, Edmonton, AB, Canada; 2 Physiology, University of Alberta, Edmonton, AB, Canada; 3 Hebrew University, Jerusalem, Israel; 4 Jimei University, Xiamen, Fujian, China; 5 HRU, Málaga, Spain; 6 Ludwig-Maximilians-Universitat Munchen, Munchen, Bayern, Germany; 7 Hospital for Sick Children, Toronto, ON, Canada; 8 Biological Science, University of Alberta, Edmonton, AB, Canada

## Abstract

**Background:**

The pathogenesis of pCD remains poorly understood, but evidence suggests roles for genetics, environment, immune response, and gut microbes. Microbial changes can contribute to chronic inflammation and correlate with disease severity. Metabolomics reflects interactions between host immune and gut microbial function by quantifying compounds in biological samples. Therefore, metabolomics provides a unique opportunity to gain insight into pCD pathogenesis.

**Aims:**

To correlate disease severity, metabolites, and clinical data by applying machine learning algorithms in pediatric Crohn Disease (pCD).

**Methods:**

ImageKids is a multicenter, prospective, cohort observational study, conducted to develop magnetic resonance enterography (MRE) indices for pCD. Paired serum specimens were collected at study initiation (Visit One; V1) and completion (Visit Four; V4; 18 months) for 120 pCD patients. Serum from patients with representative clinical scenarios and paired samples was analyzed at The Metabolomics Innovation Centre (TMIC; University of Alberta) and 131 metabolites were identified. Metabolites were analyzed via Unsupervised (U.ML) and Supervised (S.ML) Machine Learning algorithms based on Scikit-learn library in Python. Principal Component Analysis (PCA) was used to identify the variation pattern of the patients’ metabolome. Classifiers and regression algorithms were trained to assess correlation with disease activity.

**Results:**

Results were available for the 56 paired samples. U.ML demonstrated distinct metabolome profiles with V1 clustering mainly attributed to aspartic acid, glutamic acid, and kynurenine. V4 clustering was mainly attributed to spermidine, spermine, total dimethylarginine. Furthermore, demographics was found as an important environmental factor driving distinct patterns of the metabolomics profile.

After training different classifiers and regressors with S. ML algorithms, metabolome data were correlated with disease severity (defined by C-reactive protein and fecal calprotectin). Isoleucine, p-hydroxyhippuric acid, and putrescine were the top three compounds associated with disease severity. The accuracy of our classification models was of 80% and the coefficient of determination of our regression models was 0.5

**Conclusions:**

Metabolomic analysis can provide insight into disease pathogenesis and help predict disease severity among pCD patients. The correlation between metabolomics and disease severity might allow a better understanding of changes in host-microbe interactions and introduce new diagnostic or therapeutic options.

**Funding Agencies:**

CIHR

